# Curve progression in non-surgically treated patients with idiopathic scoliosis: a cohort study with 40-year follow-up

**DOI:** 10.2340/17453674.2024.42659

**Published:** 2025-01-20

**Authors:** Casper DRAGSTED, Lærke RAGBORG, Søren OHRT-NISSEN, Thomas ANDERSEN, Martin GEHRCHEN, Benny DAHL

**Affiliations:** Spine Unit, Department of Orthopaedic Surgery, Rigshospitalet, Copenhagen, Denmark

## Abstract

**Background and purpose:**

Treatment of idiopathic scoliosis in childhood aims to prevent curve progression. It is generally accepted that curves > 50° have the highest risk of progression, but less well described is what happens with mild to moderate curves. The aim of this study was to assess long-term curve progression and health-related quality of life (HRQoL) and compare thoracic and thoracolumbar/lumbar (TL/L) curves.

**Methods:**

We identified 177 patients diagnosed with a pediatric spinal deformity and treated at our institution from 1972 through 1983. 91 of 129 eligible patients with idiopathic scoliosis completed follow-up (71%). Patient files from treatment/observation in childhood were reviewed including detailed descriptions of radiographs. At follow-up we assessed long standing full-spine radiographs and HRQoL with the Scoliosis Research Society 22 revised questionnaire.

**Results:**

Mean follow-up was 41 years (standard deviation [SD] 2.5 years). 21 patients underwent surgery in adolescence or early adulthood leaving 70 patients for analysis of curve progression, of whom 61 had complete radiographs. For patients with a main curve < 25° at the end of treatment in adolescence (n = 19) mean curve progression was 7° (SD 9); for 25–40° curves (n = 26) 16° (SD 13); for 40–50° curves (n =10) 22° (SD 8); and for curves > 50° (n = 6) 17° (SD 6). There was a linear association between main curve size at follow-up and SRS-22r subtotal score (P = 0.003).

**Conclusion:**

We found substantial curve progression for patients with main curves > 25° at end of treatment, but with a considerable variation between patients. Curve progression was not associated with curve size at the end of treatment and did not differ significantly between thoracic and TL/L curves. Larger main curve size at follow-up was associated with lower HRQoL.

The natural history of idiopathic scoliosis has been examined in a few studies [1-3], showing that the scoliosis can progress to more severe deformities causing pulmonary compromise and long-term disability [4-6]. Therefore, the primary goal of treatment is to prevent curve progression by either bracing or surgical treatment. It is generally accepted that curves > 50° will continue to progress throughout adulthood [2,7-9], and these patients are recommended surgery according to international guidelines. However, the long-term outcome in mild to moderate curves after the end of treatment is not established. These patients are usually followed until skeletal maturity and then left with no further follow-up.

Previous studies with 20–25 years’ follow-up have shown limited curve progression in brace-treated patients and only slightly lower health-related quality of life (HRQoL) compared with controls [4,9-12]. The question is what happens if the scoliosis continues to progress later in adulthood when degenerative changes to the spine usually occur and whether this leads to increased pain and disability. Although several factors are considered in the decision for surgery in childhood, the Cobb angle of the major curve is still the main measure to guide treatment. Current knowledge on long-term curve progression is mainly from studies of untreated patients before the introduction of modern brace and surgical treatment.

Therefore, the primary aim of our study was to examine long-term curve progression in nonoperated patients with idiopathic scoliosis and assess how curve progression is related to curve size at skeletal maturity, and, second, compare curve progression between main thoracic and main thoracolumbar/lumbar (TL/L) curves and assess whether long-term HRQoL is related to curve size and curve progression.

## Methods

### Patients

**Figure 1:**
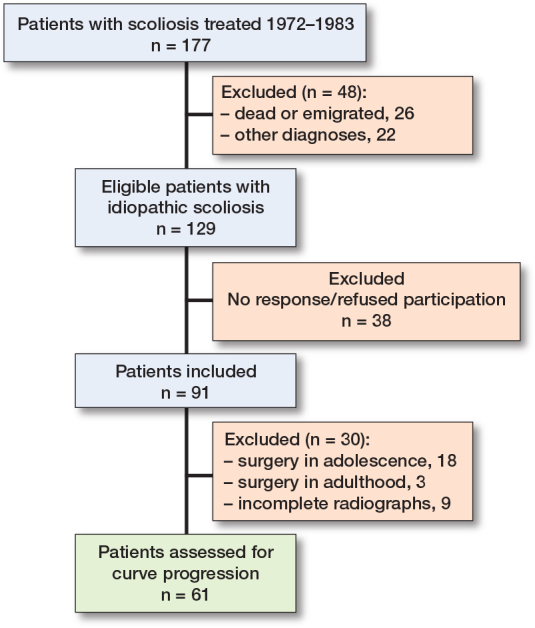
Flowchart of patients.

The study was conducted and reported according to STROBE guidelines. From a local archive, we identified 177 patients with scoliosis treated at our institution in the years 1972–1983 (Figure 1). The archive contained index cards with patient identification numbers and basic information on diagnosis, treatment, and appointments at the clinic. We retrieved the patient files from childhood to confirm the diagnosis. From these, we obtained information on basic demographics, age at diagnosis, and treatment (brace type, brace duration, surgical procedures). We excluded patients with infantile, neuromuscular, syndromic, and congenital scoliosis. Patients were generally treated according to modern guidelines with brace initiation for curves > 25° and spondylodesis for progressive curves > 50°. Some patients were observed only, either due to having a mild scoliosis or due to a late diagnosis after the growth spurt.

All eligible patients were contacted by mail with a second reminder by mail or phone and invited to a clinical follow-up visit.

### Parameters

All patients underwent a clinical examination and full-spine standing radiographs. Examinations included a clinical assessment of the patient’s deformity and questions regarding work and educational status, conservative treatment, and surgery in adulthood. Prior to the follow-up visit, patients responded to validated HRQoL questionnaires: the Scoliosis Research Society 22 revised (SRS-22r) and EuroQol 5-dimension 3-level (EQ-5D) [13]. Overall HRQoL measures and work status have been reported in a former study and compared with a representative background population [14].

### Radiographic assessment

Radiographs from the treatment in childhood were not available. However, the radiographs were described in detail in the patient files by the treating surgeon and a radiologist including information on curve type, location, and magnitude. For comparison with former studies on natural history [2] and according to current treatment guidelines, we divided patients into 4 groups according to curve size at the end of treatment/observation in adolescence: mild (main curve < 25°); low risk moderate (main curve 25–40°); high risk moderate (main curve 40–50°); and severe curves (main curve > 50°). Curves with apex above Th12 were defined as thoracic and curves with apex at Th12/L1 and below were defined as thoracolumbar/lumbar (TL/L) curves. The latest registered main curve size in adolescence was defined as curve size at end of the treatment.

At the clinical follow-up, we obtained standing frontal and sagittal full-spine radiographs. These were uploaded to and analyzed in the validated online imaging software KEOPS (SMAIO, Lyon, France) [15]. We measured the Cobb angles of the main curve and secondary curve. Main curve at follow-up was decided by the main curve location in adolescence, and curve progression was calculated as the difference between main curve at the end of treatment in adolescence and main curve at follow-up. Secondary curves were inconsistently described in the patient files; hence progression of secondary curves could not be assessed.

### Statistics

Statistical analyses were performed in R (version 4.1.2; R Foundation for Statistical Computing, Vienna, Austria). Data was assessed with histograms and presented in scatterplots. Normally distributed results were reported as mean with standard deviation (SD). SRS-22r data was non-normally distributed and reported as median with interquartile range (IQR). Normally distributed outcome data were compared using non-paired Student’s t-test, and non-normally distributed data compared using Wilcoxon rank sum test and Kruskal–Wallis test in comparison of more than 2 groups. A P value < 0.05 was considered significant. We used linear regression analyses to assess associations between curve size at the end of treatment and curve progression. Linear model assumptions were examined with residual plots and the model fit was expressed with R^2^ values. To explore the association between moderate to severe curves and curve progression, we did a sensitivity analysis by plotting only patients with curves above 25° at the end of treatment against curve progression. Linear regression was also applied to assess the relationship between curve size and long-term HRQoL measured with SRS-22r domains and subtotal score. For all models we explored different models including a polynomial regression model for a better curve fit and these were compared with linear regression models with the partial f-test.

### Ethics, registration, data sharing, use of AI, funding, and disclosures

Written informed consent was obtained from all participating patients. The study was approved by the regional committee on health research ethics (ref: H-18000884) and the local data protection authorities (ref: P-201^9^-782). This manuscript is entirely original and was written with no use of artificial intelligence. The study was carried out without specific funding and the authors have no disclosures related to this study. Complete disclosure of interest forms according to ICMJE are available on the article page, doi: 10.2340/17453674.2024.42659

## Results

91 patients were included of 129 eligible patients with juvenile or adolescent idiopathic scoliosis, corresponding to a follow-up rate of 71%. Of these, 18 patients underwent Harrington rod instrumentation in adolescence and a further 3 patients were operated on in young adulthood, leaving 70 patients for analysis of curve progression. 6 patients lacked information on curve size at the end of treatment in their old patient files and were referred to as having for example “minor curve” or “moderate curve.” A further 3 patients did not have radiographs performed at follow-up primarily due to long travel distance to the clinic. This resulted in calculation of exact curve progression for 61 patients (Figure 1).

There was no statistically significant difference in baseline characteristics (age, sex, treatment, curve type, and severity) between included patients and eligible patients not included in the study. Patients were mainly female (97%) with a mean age at diagnosis of 13.8 (SD 1.9) years. 41 (61%) patients were treated with a Boston brace in adolescence, and the remaining patients were observed. Mean age at the clinical follow-up visit was 54 (SD 2.6) years, corresponding to a mean follow of 41 (SD 2.5) years. Main curve size at follow-up for the whole population was an average of 45° (SD 21), ranging from 6–80°.

For patients with a main curve < 25° at the end of treatment in adolescence, mean curve progression was 7° (SD 9); for patients with a main curve of 25–40° mean curve progression was 16° (SD 13); for patients with a main curve of 40–50° mean curve progression was 22° (SD 8); and for patients with a main curve > 50° mean curve progression was 17° (SD 6) (Table 1). In brace-treated patients, mean curve progression from brace cessation to last follow-up in adolescence was 1° with only 5/41 patients (12%) progressing > 5°.

**Table 1:** Curve progression according to curve size at the end of treatment in adolescence. Values are mean with (SD) or count

Factor	Mild (< 25°) (n = 19)	Low-risk moderate (25–40°) (n = 26)	High-risk moderate (40–50°) (n = 10)	Severe (> 50°) (n = 6)
At follow-up				
Main curve, °	22 (12)	32 (4)	65 (7)	71 (6)
Secondary curve, °	16 (4)	32 (12)	41 (21)	57 (11)
Curve progression, °	7 (9)	16 (13)	22 (8)	17 (6)
Yearly curve progression, °/year	0.2 (0.2)	0.4 (0.3)	0.5 (0.2)	0.4 (0.1)
Patients with curve progression				
> 10°, n	8	15	10	4
> 20°, n	1	10	6	1

**Figure 2:**
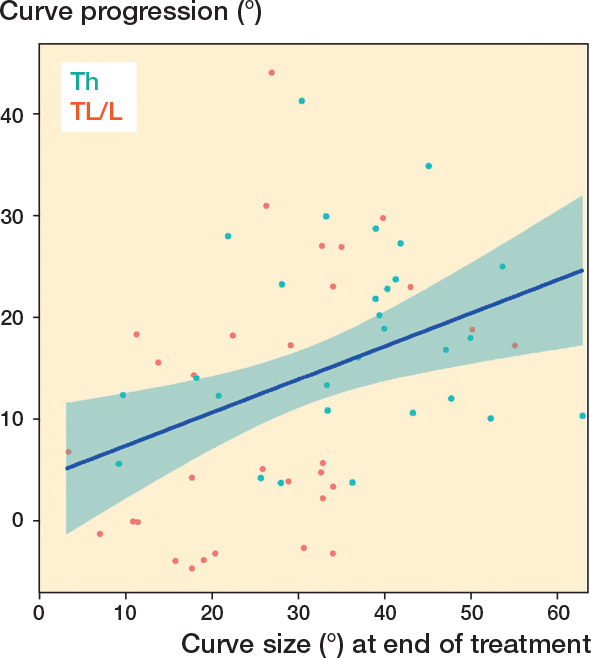
Curve progression plotted against main curve size at the end of treatment in adolescence.A linear regression line with 95% confidence intervals is shown (blue line), P = 0.003 for the linear regression and R2 = 0.14 for the model fit. Th = thoracic; TL/L = thoracolumbar/lumbar.

**Figure 3:**
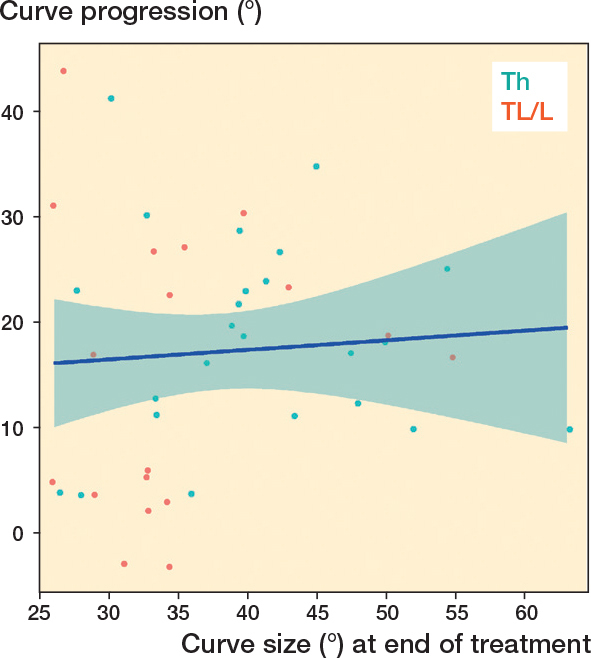
Curve progression plotted against main curve size at the end of treatment in adolescence, only curves > 25°. A linear regression line with 95% confidence intervals is shown (blue line), P = 0.7 for the linear regression and R2 = 0.005 for the model fit. Th = thoracic; TL/L = thoracolumbar/lumbar.

For the total cohort we found a large variation in curve progression and only a slight linear association (P = 0.003, 95% confidence interval [CI] for the regression coefficient 0.11–0.54) between main curve size at the end of treatment and curve progression (Figure 2). The R^2^ value for the linear model fit was only 0.14. For curves > 25° at the end of treatment, there was no linear association with curve progression (P = 0.7, CI for the regression coefficient –0.32 to 0.50) (Figure 3).

We found a higher curve progression for patients with a main thoracic curve compared with patients with a main TL/L curve (Table 2); mean difference 7°, CI 1–12° (P = 0.03). Patients with a main thoracic curve also had an overall larger main curve size at the end of treatment; mean difference 9°, CI 2–16° (P = 0.009). For patients with main curves > 25° at the end of treatment, there was no statistically significant difference in curve progression; mean difference 3°, CI –4 to 11° (P = 0.4).

**Table 2:** Comparison between thoracic and thoracolumbar/lumbar (TL/L) main curves. Values are mean degrees with (SD)

Factor	Thoracic (n = 30)	TL/L (n = 31)	P value
Main curve at the end of treatment	35 (13)	26 (12)	0.009
Main curve at follow-up	53 (18)	37 (21)	0.003
Secondary curve at follow-up	33 (19)	31 (15)	0.6
Curve progression	18 (10)	11 (13)	0.03
Curve progression for moderate/severe curves (> 25°)	19 (10)	15 (14)	0.4

**Figure 4:**
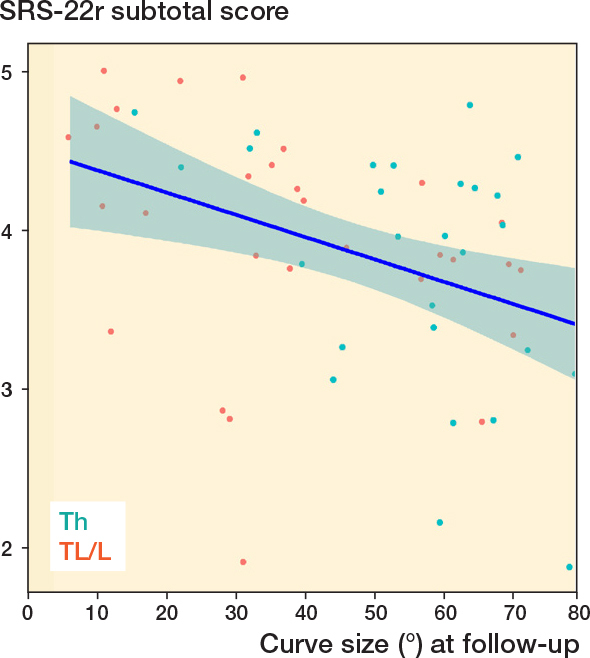
Scoliosis Research Society 22 revised (SRS-22r) subtotal score plotted against main curve size at follow-up. A linear regression line with 95% confidence intervals is shown (blue line), P = 0.003 and R2 = 0.15 for the model fit. The regression coefficient is –0.014, corresponding to a 0.14 reduction in SRS-22r subtotal score for every 10° increase in main curve size. Th = thoracic; TL/L = thoracolumbar/lumbar.

There was no linear association between curve progression and long-term HRQoL expressed with SRS-22r subtotal score (P = 0.1), regression coefficient –0.013, CI –0.039 to 0.004. However, there was a linear association between curve size at follow-up and SRS-22r subtotal score (P = 0.003) and the subdomains function (P = 0.01), pain (P = 0.005), and self-image (P < 0.001), but not for the mental health subdomain (P = 0.5). For the SRS-22r subtotal score, the regression coefficient was –0.014, CI –0.023 to –0.005, corresponding to a 0.14 reduction in SRS-22r subtotal score for every 10° increase in main curve size at follow-up (Figure 4).

SRS-22r scores relative to the main curve size at the end of treatment in adolescence are indicated in Table 3. The scores differed statistically significantly between groups for all domains besides mental health, and with lower scores for patients with larger curves.

**Table 3:** Scoliosis Research Society-22r (SRS-22r) scores according to curve size at the end of treatment in adolescence. Values are median with (interquartile range)

Factor	Mild (< 25°) (n = 19)	Low-risk moderate (25–40°) (n = 26)	High-risk moderate (40–50°) (n = 10)	Severe (> 50°) (n = 6)	P value
Function	4.8 (4.5–4.9)	4.4 (3.6–4.6)	3.8 (3.2–4.4)	3.3 (2.8–4.4)	0.04
Pain	4.4 (4.0–4.9)	3.6 (3.0–4.4)	3.7 (2.4–4.2)	3.2 (2.1–3.7)	0.02
Self-image	4.2 (3.7–4.8)	3.6 (3.0–4.0)	3.6 (2.7–3.7)	3.1 (2.8–3.3)	0.007
Mental	4.4 (4.0–4.8)	4.4 (4.0–4.4)	4.3 (3.8–4.6)	3.9 (3.8–4.4)	0.7
Subtotal score	4.4 (4.2–4.7)	3.8 (3.5–4.3)	3.9 (3.2–4.2)	3.5 (2.9–4.0)	0.003

## Discussion

In this study of a representative cohort of patients with idiopathic scoliosis with more than 40 years’ follow-up, we found substantial curve progression for moderate curves. Our results indicate that curve progression itself is not decisive for long-term HRQoL; however, a larger main curve at follow-up is associated with lower HRQoL measured with the SRS-22r questionnaire.

Previous follow-up studies of brace-treated patients with 20–25 years’ follow-up have shown only minor curve progression of 5–8° [9,11,16,17]. Comparably, Ohashi et al. [7] reported a yearly progression of 0.41 in TL/L main curves. Similar to our results, the studies show considerable variation in the reported curve progression between patients. In the study by Danielsson et al., 36% of the patients progressed more than 10° [9]. In our study we did not have radiographs available throughout adulthood, and so we cannot tell when curve progression occurs. However, the reported curve progression cannot be explained by progression following brace cessation. Our results show that curve progression continues for some patients and might even be accelerated later in adulthood.

Mean progression in this study is biased, as some of the patients with high risk of progression have been selected for spinal fusion in adolescence or early adulthood in accordance with former studies [17,18]. And, with only 6 patients with a main curve of > 50° at the end of treatment, it is not appropriate to draw any firm conclusions regarding this group. Nevertheless, our results suggest that for moderate sized curves there is no association between end-treatment curve size and long-term curve progression. Curve progression varied considerably between patients with moderate to severe curves. 2 patients had progressed more than 40° at follow-up, both with a curve of 25–30° at the end of treatment but with different curve locations. Follow-up radiographs showed a balanced double curve for both patients. This underlines the difficulties in predicting curve progression for the individual patient based on this data.

Our results are in line with recently published studies showing considerable long-term progression for patients with moderate curves [19,20]. Some of these patients could profit from spinal fusion to prevent substantial curve progression.

Contradictory results have been presented regarding curve location and long-term progression. Older studies of untreated patients show higher curve progression for thoracic main curves [2,9], whereas more recent studies suggest progression in main TL/L curves [7,17]. We found a higher curve progression for thoracic main curves. However, the difference seems to be explained partly by more patients with a mild TL/L curve in our cohort, as also illustrated by a larger mean Cobb angle at the end of treatment in patients with thoracic curves. When looking only at curves > 25°, there was no statistically significant difference in curve progression between thoracic and TL/L main curves.

The results suggest that main curve size at follow-up is associated with lower HRQoL but not curve progression in itself. We have previously shown that long-term overall HRQoL in patients with idiopathic scoliosis is slightly lower than that of the background population [14]. Mid-term follow-up results regarding HRQoL in brace-treated patients have been more inconsistent but with overall satisfactory results. At mid-term follow-up patients are in their early decades of adulthood with a more flexible spine and compensatory mechanisms still intact. This might contribute to the good outcomes in terms of HRQoL measurements. But as the patients become older than 50 years of age, degenerative changes to the spine might contribute to worsening of the deformity. Also, the scoliosis might accelerate pain and disability caused by degenerative changes that would not otherwise cause problems.

### Limitations

Examining HRQL data retrospectively at long-term follow-up is possibly affected by regression towards the mean; however, it is impossible to tell whether this effect differs with curve severity thereby systematically biasing the results. Sample size issues are inherent in this type of study and limit the statistical power. In addition, as spinal fusion had been performed in the majority of patients with curves of more than 50°, we are unable to draw any firm conclusions regarding this group. Likewise, patients lost to follow-up might introduce selection bias in the results. Though we cannot account for the consecutiveness of the cohort, we find the population in this study very representative, ranging from patients with mild scoliosis to patients with very severe curves and different curve locations. Moreover, a 71% follow-up rate is high considering the more than 40 years’ follow-up. It is one of the largest long-term follow-up studies including radiographic assessment and patient-reported outcomes.

### Conclusion

We found a substantial curve progression for patients with main curves > 25°at the end of treatment in adolescence. Curve progression was not related to curve size at the end of treatment but varied considerably among patients. Long-term HRQoL was not associated with curve progression; however, a larger main curve size at follow-up was associated with lower SRS-22r scores.

*In perspective,* our results indicate that patients are not “home safe” with a curve below 50° at the end of treatment. It adds important information in the guidance of patients and parents in their decision for treatment. We should possibly follow patients for longer than only until skeletal maturity to identify patients with progressive curves and discuss surgery with the patient to prevent long-term consequences of further progression of their deformity. This could be in the form of a clinical examination and radiographic control 5 years into early adulthood for patients with moderate curves. Moreover, this would allow us to collect information on curve progression after skeletal maturity. This would be in line with studies suggesting that curve progression is not only related to growth and for some patients continues after skeletal maturity [21].

## Notes

CD: project planning, study design, data acquisition, data analysis, manuscript writing, and manuscript revision. LR: data acquisition, data analysis, manuscript revision, and approval. SO: study design, data analysis, manuscript revision, and approval. TA: project planning, manuscript revision, and approval. MG: project planning, study design, manuscript revision, and approval. BD: project planning, study design, manuscript revision, and approval.

Handling co-editors: Paul Gerdhem and Philippe Wagner

*Acta* thanks René M Castelein, Elias Diarbakerli, and Thomas Johan Kibsgard for help with peer review of this manuscript.
